# Do Rates of Mental Health Symptoms in Currently Competing Elite Athletes in Paralympic Sports Differ from Non-Para-Athletes?

**DOI:** 10.1186/s40798-021-00352-4

**Published:** 2021-08-24

**Authors:** Lisa S. Olive, Simon Rice, Matt Butterworth, Matti Clements, Rosemary Purcell

**Affiliations:** 1 Orygen, 35 Poplar Road, Parkville, Melbourne, Victoria 3052 Australia; 2 Centre for Social and Early Emotional Development, School of Psychology, Deakin University, Geelong, Australia; 3 IMPACT Institute for Mental and Physical Health and Clinical Translation, Deakin University, Geelong, Australia; 4 The Centre for Youth Mental Health, University of Melbourne, Melbourne, Australia; 5 Australian Institute of Sport, Athlete Wellbeing and Engagement, Canberra, Australia

**Keywords:** Mental health, Elite athlete, Paralympic sports, Depression, Anxiety, Body dissatisfaction, Alcohol use, Elite sport, Athlete

## Abstract

**Background:**

This study addresses the lack of comparative data on the mental health of athletes in Paralympic sports (‘para-athletes’) and non-para athletes by examining the prevalence and correlates of mental health symptoms in a national sample of elite athletes representative of the population from which it was drawn on age and para-status.

**Methods:**

A cross-sectional, anonymous, online-survey was provided to all categorised (e.g. highest level) athletes, aged 17 years and older, registered with the Australian Institute of Sport (*n* = 1566). Measures included psychological distress, mental health caseness, risky alcohol consumption, body weight and shape dissatisfaction, self-esteem, life satisfaction, and problem gambling. Correlates of outcomes included individual (e.g. demographic and psychosocial) and sport-related variables.

**Results:**

The participation rate was 51.7% (*n* = 810), with valid data available from 749 athletes. No significant differences were observed between athletes from para- and non-para-sports on most mental health symptoms, with the exception of alcohol consumption (*p* < .001) and self-esteem (*p* = .007), both lower in athletes from para-sports. A trend for an interaction was found for anxiety and insomnia (*p* = .018), whereby the difference between athletes from para- and non-para-sports was qualified by gender.

**Conclusions:**

In a large sample of elite athletes, mental health and wellbeing symptoms are comparable between athletes from para- and non-para-sports, with the exception of para-athletes reporting lower alcohol consumption but also lower self-esteem. While overall mental health and wellbeing symptom profiles are largely similar, attention to areas of differences will help to better address the unmet and distinct mental health needs of athletes from para-sports.

## Key Points

- Minimal differences in mental health and wellbeing symptoms were observed between a nationally representative sample of elite athletes from para- and non-para-sports.
- Of these minimal differences, athletes from para-sports reported lower alcohol consumption but also lower self-esteem compared to athletes from non-para-sports.
- The findings reported here highlight a similar need for mental health supports across athlete populations.

## Introduction

There is increasing recognition of the mental health challenges and needs of elite and professional sportspeople [1]. However, there is little empirical evidence regarding the rates of mental health symptoms among elite athletes in Paralympic sports (hereon termed ‘para-athletes’) and a lack of comparative data with athletes from non-para-sports. To date, meta-analyses that estimate the prevalence of mental health symptoms in elite athletes, ranging from 19% for general psychological distress and/or alcohol misuse, through to 34% for anxiety/depression [2, 3], have mostly involved athletes from non-para-sports (e.g. predominantly male, professional team sports).

The existing body of knowledge regarding mental health in elite athletes applies to athletes from para-sports; however, para-athletes are likely to experience a range of additional impairment-specific stressors that have the potential to compromise their mental health, such as discrimination [4], a lack of sufficient, adaptive sport facilities, logistical challenges in travel to competition sites and the cost of impairment-specific equipment [5]. Conversely, the Paralympic Movement and the status of the Paralympics has potentially facilitated the development of a range of protective factors for athletes relating to their involvement in elite sport and their identity as an elite athlete, which in turn may positively affect mental health [6, 7]. Understanding mental health symptoms, as well as mental *wellbeing*, in para-athletes is necessary to inform appropriate response frameworks and interventions.

In a systematic review (12 included studies) comparing general well-being in Olympic and Paralympic athletes [8], two studies reported significant differences in favour of Olympic athletes for life satisfaction, mood-disturbance, fatigue and depression [9, 10]. A more recent narrative review that focused specifically on Paralympic athletes concluded that, given the paucity of published data much of which has been qualitative by design, relied on small sample sizes and often non-standardised measures of psychopathology, the only definitive course is for more focused research on the mental health of those who participate in elite competitive para-sports, including comparisons with elite athletes from non-para-sports [11].

To address this gap, we compared the prevalence and correlates of mental health symptoms in elite para-athletes with the rates among athletes from non-para-sports in a national sample of athletes representative of the population from which it was drawn on age and para-status (the Australian Institute of Sport [AIS] high performance sporting system). As the rates of mental health symptoms in the general (e.g. non-athletic) population are reported to be higher among people with disabilities than in non-disabled individuals, particularly for depression [12], we hypothesised that para-athletes would report higher rates of mental health symptoms compared to athletes from non-para-sports.

## Methods

### Participants

All elite para- and non-para-athletes aged 17 years and over, who were supported by the AIS via being contracted with a national sporting organisation (NSO) were invited to participate in an anonymous, online survey regarding their mental health and wellbeing (see [13] for a detailed study overview). AIS supported athletes can receive access to a range of resources, which include but are not limited to monetary support in the form of a grant, access to training/high performance resources (technology, equipment and personnel) and access to mental health and wellbeing support. There were no exclusion criteria other than age. Athletes completed the survey between July and September 2018. All participants were provided with information about the purpose of the survey prior to commencing and informed consent was obtained from athletes by them choosing to commence the survey, which was explained in the information provided to participants. The University of Melbourne Human Ethics Research Committee approved the study (#1442705).

### Survey Items

The survey consisted of the following sections and scales (see [13], for a comprehensive overview of the survey items).

#### Background Information/Demographics

In addition to basic demographic characteristics, athletes were asked whether or not they had competed in the 2018 Winter Olympics and Paralympics, or the 2018 Commonwealth Games (which included para-sports); their sport type (individual or team-based); the number of years contracted as an NSO athlete; whether they were currently injured or in adapted training programme due to injury; the frequency of sport-related travel over the past year; the experience of missing significant life events due to travel for sport; and whether they had ‘ever been treated for a psychological issue or mental health problem (e.g. anxiety, depression)’.

#### Symptom Outcome Measures

Outcome measures were included in the following order, and where measured over the 4 weeks prior to the survey unless otherwise indicated. *Mental health symptoms* and *‘caseness’* were assessed using the 28-item General Health Questionnaire (GHQ-28) [14], which yields a total score and scaled scores for (i) somatic complaints, (ii) anxiety and insomnia, (iii) social dysfunction, and (iv) severe depression (including suicidal ideation). ‘Probable caseness’, defined as symptoms that adversely affect quality of life and are of a level frequently found among individuals seeking help from health professionals [15], can also be calculated from the GHQ-28. *General Psychological Distress* was measured using the Kessler 10 (K-10), a 10-item screening tool assessing psychological distress, including nervousness, fatigue, hopelessness, and depression [16]. The K-10 has been widely validated in a range of populations [17–19] and demonstrated good reliability in the current sample of elite athletes (*α* = 0.89). *Self-esteem* was measured using the validated 10-item Rosenberg Self Esteem Scale [20], with responses provided on 4-point Likert scale (1 = strongly disagree to 4 = strongly agree), whereby higher scores indicate higher self-esteem. This scale was found to be reliable in the current sample (*α* = 0.87). *Mental wellbeing* was measured using the Warwick-Edinburgh Mental Wellbeing Scale (WEMWBS) [21], a 14-item scale assessing positive aspects of mental health as a single factor, such as feeling useful, relaxed, and optimistic [21, 22]. Responses are measured on a 5-point Likert scale (1 = none of the time to 5 = all of the time), with higher scores indicating greater mental wellbeing. The WEMWBS showed good reliability in the current sample (α = 0.93). *Gambling behaviour* over the past 12 months was measured using the 3-item Problem Gambling Severity Index (PGSI) [23]. Responses are provided on a 5-point Likert scale (ranging from ‘never’ to ‘always’), with higher score indicating more problematic gambling behaviour. The PGSI showed good reliability in the current sample (*α* = 0.94). *Maladaptive response to psychological distress* was measured using two subscales from the Male Depression Risk Scale (MDRS) [24], namely the anger and aggression (4 items) and risk-taking behaviour (3 items) subscales. Participants rate items relative to the preceding month, where 0 = not at all to 7 = almost always, with higher scores indicating greater risk. Both subscales were shown to be reliable in the current sample (anger and aggression *α* = 0.90; risk-taking behaviour *α* = 0.80). *Body weight and shape dissatisfaction* were assessed using the weight (5 items) and shape (8 items) subscales from the Eating Disorders Examination Questionnaire (EDEQ) [25]. Participants rate the severity of the psychopathology of eating disorders on a 7-point Likert scale, ranging from 0 = absence of the feature to 6 = feature present to an extreme degree, with higher scores indicating greater severity. *Alcohol use* in the past 12 months was measured by the Alcohol Use Disorders Test (AUDIT) [26], which yields a total score derived from scaled scores for (i) alcohol consumption, (ii) alcohol dependence, and (iii) alcohol-related problems, whereby higher scores indicate higher risk of an alcohol use disorder. The AUDIT total scale was found to have acceptable reliability in the current scale (*α* = 0.69). *Satisfaction with life* (no time limit) was assessed using the 5-item Satisfaction with Life Scale [27]. Each item is assessed on a 7-point Likert scale ranging from 1 = strongly disagree to 7 = strongly agree, with higher scores indicating higher life satisfaction, and was shown to be reliable in the current sample (*α* = 0.90). *Quality of life* was assessed using a single item that rated quality of life on a 5-point scale, ranging from ‘very poor’ to ‘very good’.

#### Psychosocial Correlates

*Adverse life events* were assessed in the 12 months prior to the survey and lifetime, given the relationship between such events and psychopathology [28]. Thirteen items were included, such as death or serious illness of a loved one, relationship breakdown, and discrimination.

*Social support* was assessed using three questions [29] that examined the perceived availability and adequacy of social support, and sources of support, and *social isolation* was measured using three items that asked participants to rate the frequency with which they felt they lacked companionship, felt left out, and felt isolated from others. *Help seeking* for a personal or emotional problem was assessed using the 10-item General Help Seeking Questionnaire [30], where participants rate on a 7-point Likert scale (1 = extremely unlikely to 7 = extremely likely) how likely it is that they would seek help. *Coping style* was assessed using the Billings and Moos Coping Scale [31], which yields scores on active-cognitive (6 items), active-behavioural (6 items) and avoidance coping (5 items). Participants endorsed whether or not they utilised each coping strategy, using a yes/no response format, with higher scores indicating greater use of that style of coping.

### Patient and Public Involvement

The survey was developed in consultation with the AIS’ Athlete Wellbeing and Engagement team, who consulted with former and current athletes in the study design.

### Data Analyses

The binary GHQ-28 ‘probable caseness’ outcome variable was analysed using logistic regression (categorical scoring involves applying weights to the four response alternatives (0-0-1-1) to produce a scoring range of 0–28. A cut-off of 5 was applied [15] in which participants scoring a total of five or more are considered a probable case). Other categorical outcomes variables, including the AUDIT Hazardous item (categorical scoring was based on AUDIT risk level classification where 0 = low risk and 1 = hazardous risk or higher) and EDEQ derived body weight and shape dissatisfaction subscales (0 = no shape or weight dissatisfaction, 1 = endorsed 1 item, 2 = endorsed both items), were also analysed using logistic regression. For all other continuous variables, a two-way analysis of variance was used to determine differences between athletes from para-sports and non-para-sports, males and females and their interaction (para-status x gender), with adjustment made for age. Given the number of analyses performed on the same dataset, outcomes were evaluated as statistically significant at *p* ≤ .01 to reduce the chance of type I error, with Cohen’s *d* reported as a measure of effect size. All analyses were conducted using SPSS Version 25.

## Results

### Participant Characteristics

A total of 1566 athletes, of whom 16.0% were para-athletes, were registered with the AIS and aged 17 years or older at the time of the survey and therefore eligible to participate (52.5% male; 47.5% female; overall mean age = 24.5 years). Of these, 810 consented to participate (51.7% response rate) and full data were available for analysis for 749 participants. The participating athletes were representative of the eligible population in relation to para-status (14.7%) and mean age (24.6 years); however, a higher proportion of female athletes completed the survey (54.1%).

The 110 participants from para-sports represented 44% of currently engaged AIS para-athletes. The majority were male with a mean age of 30.71 (SD = 10.67), and Australian-born (91.8%). At the time of the survey, most athletes from para-sports (74.7%) reported being engaged in employment in addition to their role as elite athlete (comprising 59.2% in paid employment, and 15.5% in unpaid voluntary work) and 32% were studying. Almost half of the participants were single or never married (49.5%) and 37% had completed their highest level of secondary education. The demographic and sports-related characteristics of the para-athletes compared to non-para-athletes are summarised in Table 1.

**Table 1:** Demographic and sports-related characteristics of the para-athletes compared to non-para-athletes

Demographic characteristics	Para-athletes % (*n*)	Non-para-athletes % (*n*)	Sport-related characteristics	Para-athletes % (*n*)	Non-para-athletes % (*n*)
Gender (% female)	43.0 (46)	57.1 (359)	Individual sport** Team-based sport	75.1 (82) 24.8 (27)	58.8 (371) 41.2 (260)
Mean age (SD)**	30.7 (10.6)	23.4 (4.7)	Recent competition	24.5 (27)	20.6 (131)
Relationship status**			*Main activity related to sport in the past month*		
Single/never married	49.5 (54)	55.0 (350)	Actively engaged in sport	49.1 (54)	57.6 (367)
Partnered	29.4 (32)	36.8 (234)	Training (in-season)	18.2 (20)	19.0 (121)
Married	18.3 (20)	7.5 (48)	Training (off-season)	20.0 (22)	12.6 (80)
Separated/divorced	2.8 (–)	0.6 (–)	Recovering from injury	9.1 (10)	8.2 (52)
			Other	3.6 (-)	2.7 (17)
*Sexual orientation* Heterosexual Bisexual Same sex attracted Other^	88.2 (97) 3.6 (–) 2.7 (–) 5.5 (6)	93.2 (593) 2.8 (18) 1.7 (11) 2.2 (14)	Currently injured Currently in an adapted training programme due to injury	10.1 (11) 12.7 (14)	12.9 (82) 15.9 (101)
Currently studying	68.2 (75)	77.7 (261)	Ever treated for a concussion	13.6 (15)	15.9 (101)
Highest level of education* Completed < year 12 Completed year 12 University degree Vocational qualification	14.7 (16) 37.6 (41) 29.4 (32) 18.3 (20)	14.8 (94) 54.2 (345) 21.4 (136) 9.6 (61)	Length of time as an NSO categorised athlete** Less than 12 months 1–3 years 4–7 years 8 or more years Do not know	10.1 (11) 32.1 (35) 26.6 (29) 22.2 (24) 9.2 (10)	16.0 (102) 37.3 (237) 19.5 (124) 11.2 (71) 13.7 (102)
Paid work in the past month* None Paid—casual Paid—part time Paid—full time Voluntary (unpaid)	25.5 (28) 25.5 (28) 16.4 (18) 17.3 (19) 15.5 (17)	35.1 (223) 30.2 (192) 15.4 (98) 13.1 (83) 6.3 (40)	Travel away due to sport ** Less than 1 month1–2 months 3–4 months 5–6 months 6 months or more	3.9 (29) 43.6 (48) 16.4 (18) 7.3 (8) 6.4 (7)	13.8 (103) 30.8 (196) 28.7 (183) 7.8 (50) 16.5 (105)
Living arrangements** At family home (of origin) Renting Own/mortgaged home Other#	47.7 (51) 23.4 (25) 28.0 (30) 0.9 (-)	49.8 (317) 31.4 (200) 11.6 (74 7.1 (45)	Missed significant life events due to role/travel	60.0 (66)	67.2 (429)
Ever treated for a mental health or psychological problem*	33.6 (37)	20.4 (130)	Personal concern for safety while travelling	7.3 (8)	9.6 (61)
Sought treatment for a mental health or psychological problem in the past 12 months	20.9 (37)	19.9 (127)			

**p < .001, *p < .01

^Includes ‘other’, ‘do not know’ and ‘do not want to say’

^#^Includes living with a host family

Group differences were observed in relation to age (*t*(734) − 6.6, *p* < .001, *d* = 0.9; higher in para-athletes), relationship status (*χ*^2^ = 8.3, *p* < .001), highest level of education (*χ*^2^ = 14.1, *p* < .01), length of time as an NSO categorised athlete (*χ*^2^ = 16.6, *p* < .01), work outside of sport (*χ*^2^ = 14.8, *p*<.01), current living arrangements (*χ*^2^ = 25.2, *p* < .001), type of sport (*χ*^2^ = 10.5, *p* < .001) and travel due to sport in the 12 months prior to the survey (*χ*^2^ = 22.0, *p* < .001).

Para-athletes reported experiencing a significantly greater number of adverse life events over their lifetime (mean = 3.9 [SD = 2.2] vs 2.8 [1.8], *t*(598) = − 4.1, *p* < .001, *d* = 0.5), in particular being more likely than non-para-athletes to report a personal injury or illness (57.8% vs 28.8%; *p* < .001), discrimination (33.9% vs 9.4%; *p* < .001), feeling undervalued (41.3% vs 26.2%; *p* < .001) and bereavement (53.2% vs 30.9%; *p* < .01). However, there were no group differences in relation to the total number of adverse events reported in the year prior to the survey. The availability of social support also did not differ significantly between groups, nor did the perceived adequacy of social support or the degree of social isolation (items related to lack of companionship, feeling left out, and isolated).

### Comparison of Elite Para- and Non-para-athletes’ Mental Health and Wellbeing Symptoms

Scores on the outcome measures for athletes from para-sports and non-para-sports are presented in Table 2. In unadjusted models, there were no significant group differences on any measure other than total score on the AUDIT, which was lower in para-athletes (*F*(1,641) = 11.34, *p* = .001). Adjusted models (adjusting for age and gender) revealed similar results, whereby athletes from para-sports reported significantly lower total scores on the AUDIT (*F*(1,625) = 25.21, *p* < .001), although this effect was small (*d* = 0.3). Post hoc analyses were conducted at both the subscale level of the AUDIT (e.g. consumption score, dependence score and alcohol-related problems score) and at the individual item level, adjusted for age and gender. At the subscale level, significant differences were observed between para- and non-para-athletes on the consumption score (*F*(1,496) = 25.29, *p* < .001, *d* = 0.2), with para-athletes reporting lower levels of alcohol consumption. Further investigation at the item level indicated that non-para-athletes reported consuming both a significantly greater number of standard drinks on a typical day when drinking (item 2; *F*(1,497) = 8.34, *p* = .004, *d* = 0.5) and would more frequently consume six or more standard drinks on one occasion (item 3; *F*(1,497) = 27.23, *p* < .001, *d* = 0.5). No differences were observed between athlete groups for the dependence score or alcohol-related problems score of the AUDIT (both *p* > .01).

**Table 2:** Mental health symptoms among currently competing elite para-athletes and non-para-athletes

Variable		Para-athletes (***n*** = 110) Mean (SD)			Non-para-athletes (***n*** = 639) Mean (SD)	
	Total	Males	Females	Total	Males	Females
*Psychological distress*: K-10 total score	16.7 (5.9)	16.7 (6.1)	16.9 (5.5)	16.3 (5.8)	15.3 (5.5)	17.3 (6.3)
*General Health Questionnaire-28 (GHQ-28)*: total score Anxiety/insomnia subscale score Somatic symptoms subscale score Social dysfunction subscale score Severe depression subscale score	20.8 (10.6) 5.6 (4.3) 5.8 (3.8) 7.4 (2.3) 1.8 (2.9)	20.8 (10.9) 5.8 (4.5) 5.6 (3.4) 7.3 (2.1) 2.0 (3.4)	21.5 (11.0) 5.6 (4.4) 6.3 (4.4) 7.6 (2.8) 2.0 (2.9)	19.4 (10.8) 5.4 (4.5) 5.4 (3.5) 7.1 (2.6) 1.4 (2.7)	16.5 (9.8) 4.1 (4.2) 4.6 (3.4) 6.7 (2.5) 1.1 (2.4)	21.6 (11.4) 6.4 (4.7) 6.1 (3.6) 7.4 (2.8) 1.7 (3.0)
*Satisfaction with life* scale: total score *Mental wellbeing* total score	25.5 (3.8) 48.9 (8.1)	25.6 (5.8) 49.2 (7.6)	25.3 (6.0) 49.1 (8.9)	26.7 (5.9) 50.1 (9.4)	27.3 (5.9) 52.4 (8.8)	26.5 (6.0) 48.6 (9.4)
*Self-esteem* total score	20.7 (4.8)	21.1 (4.8)	20.3 (5.4)	21.6 (4.7)*	22.7 (4.5)	20.9 (4.8)
*Male depression* rating scale total score Anger and aggression subscale score Risk-taking subscale score *AUDIT* Total score	5.1 (6.9) 2.7 (4.2) 2.4 (3.6) 2.7 (3.1)	5.9 (7.3) 3.2 (4.5) 2.6 (3.8) 3.6 (3.5)	4.5 (6.7) 2.4 (4.2) 2.1 (3.2) 1.8 (2.2)	6.1 (7.2) 3.2 (4.5) 2.9 (3.5) 4.2 (4.0)**	6.1 (7.0) 3.1 (4.2) 2.9 (3.5) 4.9 (3.5)	6.6 (7.7) 3.6 (4.9) 3.0 (3.7) 3.8 (3.8)
Alcohol consumption subscale score	3.9 (1.5)	3.4 (1.8)	2.0 (1.3)	4.3 (1.8) **	4.3 (2.3)	3.3 (1.7)
Alcohol dependence subscale score	0.4 (0.7)	0.5 (0.7)	0.3 (0.7)	0.5 (1.0)	0.5 (1.1)	0.4 (1.0)
Alcohol-related problem subscale score	1.7 (1.6)	1.8 (1.7)	1.1 (0.9)	1.3 (1.9)	1.3 (1.8)	1.3 (2.1)
	**%**	**%**	**%**	**%**	**%**	**%**
*Quality of life*: ‘good’ and ‘very good’ categories	79.3	84.6	71.1	81.0	85.5	77.7
*GHQ-28*: scoring at or above ‘caseness’ threshold	37.7	37.3	38.6	34.5	24.7	40.1
*EDE-Q* ‘high’ body weight dissatisfaction	25.3	17.6	34.2	25.8	16.3	33.3
*EDE-Q* ‘high’ body weight and shape dissatisfaction	26.7	21.6	44.8	26.2	23.1	39.7
*Problem Gambling* Severity Index: non-problem gambling category	66.7	60.0	83.3	67.5	58.1	89.2
*Hazardous alcohol consumption* threshold (AUDIT)	13.0	21.2	2.6	15.7	20.6	12.2

**p < 0.001, *p < 0.01 for adjusted models

Also in adjusted models, athletes from para-sports reported lower scores on the measure of self-esteem compared to non-para-athletes (*F*(1,683) = 7.22, *p* = .007; *d* = 0.2), and there was a similar trend for the subscale for severe depression on the GHQ-28 (which includes items on suicidal ideation) but this was not significant at the more stringent significance level (*p* = .022).

### Interaction Effects

No significant (*p* < .01) interaction effects were found between para-athlete status and gender for any included measures. There was a trend for an interaction for GHQ anxiety/insomnia, with male athletes from para-sports scoring higher on anxiety/insomnia than males from non-para-sports, and conversely females from non-para-sports reporting higher anxiety/insomnia than females athletes from para-sports (*F*(1,699) = 5.64, *p* = .018).

## Discussion

In this representative and national sample of elite athletes from para- and non-para-sports, minimal differences in mental health and wellbeing symptoms were observed between the athlete groups, with the exception of alcohol consumption and self-esteem, both being significantly lower in para-athletes. The few differences observed between the athlete groups is somewhat inconsistent with literature from the general population, where it has been suggested that the rates of mental ill-health, specifically depression, among the general population of people with disabilities are higher than in individuals without an impairment [12, 32–36]. It may be that a range of strengths-based cognitive, behavioural, and affective characteristics and qualities previously identified in para-athletes, including increased social integration, positive social-support networks, autonomy, and independence gained through sport [6, 7], are related to the current findings. Overall, our results counter what has been deemed a problematic and discriminatory assumption, that disability is inevitably associated with mental health symptoms or disorders [37, 38].

### Mental Health and Wellbeing Symptoms in Para-athletes

Para-athletes reported significantly lower alcohol consumption than non-para counterparts, which is consistent with the pattern observed in non-athletic populations, where lower rates of alcohol use and misuse are found among people with learning disabilities compared to non-disabled peers (see [39] for a review). Less is known about the prevalence and potential aetiologies for alcohol use in other forms of impairment in the general population, and given the paucity of literature on alcohol use among para-athletes, it is difficult to draw conclusions as to the context for the current results. In the current cohort, the differences in alcohol use were explained by greater alcohol consumption among non-para-athletes, rather than differences in alcohol dependence or alcohol-related problems (e.g. personal feelings of guilt/remorse, alcohol-related injury to self/others), which were low among both groups.

The results also demonstrated that para-athletes report comparatively lower levels of self-esteem. This finding is consistent with earlier work that reported significantly higher levels of global self-esteem in Flemish Olympic athletes than Paralympic athletes [40]; although this smaller study consisted of few female athletes (*n* = 3), none of whom were Paralympians. A possible explanation for this difference may relate to a number of impairment-related factors that may also affect global self-esteem, including being ‘misclassified’ or assigned to the wrong impairment category for competition [41], or negative coaching behaviours [5, 42], which is an avenue for exploration in future research.

Our results also indicated that para-athletes reported higher scores on the GHQ-28 subscale for severe depression, which include four items related to suicidal ideation, than non-para-athletes. Despite not being statistically significant given our stringent alpha, this result requires further research attention, particularly in light of the observation that 1 in 3 para-athletes (37%) in the current sample met the threshold for probable caseness on the GHQ-28, indicating symptoms at a level that would usually warrant a need for professional healthcare. Repeat studies in subsequent samples of para-athletes are required to determine if this finding is replicated, particularly given prior evidence to indicate a greater tendency for depressive symptomology among para-athletes [9, 43] and those with an impairment in the general population [12, 32–35].

A number of significant differences were observed between para- and non-para-athletes on a range of correlates, which may have served as protective factors to mental health symptoms among athletes from para-sports. On average, athletes from para-sports were *significantly older* (31 years vs 23 years; higher age has been shown to be a protective factor for mental health morbidity [44]); *spent more time as an NSO categorised athlete*, which may represent greater career longevity and stability in terms of support provided from their sport; *reported less travel*, minimising their time away from home and their usual social supports; and were *more likely to have received treatment for a mental health problem*, which may be reflective of help-seeking behaviour. Greater matching of para- and non-para-samples in future research will be important to understand whether the pattern of results reported here is replicated. The proportion of athletes from para-sports in paid employment in the current sample (59.2%) is higher than that reported in individuals with an impairment in the general population (48%) [36] and para-athletes participated in significantly more voluntary unpaid work than non-para-athletes. Athletes from para-sports have described the positive emotions experienced when ‘giving back’ to the community, through helping change people’s perceptions about impairments and being role models for other people with impairments [6]. These experiences have in turn been described by para-athletes as benefiting their sense of social integration, feelings of self-acceptance and providing a sense of purpose outside sport [6].

### Clinical Implications

Our findings highlight a similar need for mental health supports across athlete populations. Understanding the prevalence and possible risk and prognostic factors of mental health symptoms specific to athlete sub-populations can greatly assist in mental health assessment, formulation and the selection and development of appropriate prevention and clinical interventions that may be most efficacious with each subgroup.

### Strengths and Limitations

The response rate for para-athletes (44%), while acceptable given the survey methodology (and consistent with or better than other surveys), limits confidence in the representativeness of the sample. The cross-sectional design also limits the ability to draw stronger conclusions regarding the contribution of correlates of observed differences in mental health outcomes between para- and non-para-athletes and whether the few observed differences between the groups are sustained over time. This study also relied on self-report, which may have introduced bias in terms of social desirability. The anonymous nature of the survey likely negated this issue to a degree, facilitating reliability in responses. The large sample is a strength, along with the use of a wide range of mental health measures and a range of correlates. This allowed for the adjustment of potentially confounding variables and the examination of a greater range of mental health constructs than has previously been reported in the literature on elite athletes. A further limitation was the inability to adapt the survey for visually impaired athletes, something that should be considered in future studies and that we were unable to collect information on impairment among para-athletes.

## Conclusions

Differences between mental health factors among athletes from para-sports and non-para-sports appear to be minimal in this large sample of Australian elite athletes. Para-athletes reported lower alcohol consumption but also lower self-esteem compared to their counterparts from non-para-sports. These findings provide new knowledge that can inform evidence-based approaches to addressing mental health problems in para- and non-para-athletes. There is an urgent need to continue to extend this work, so that efforts to improve mental health prevention, early intervention and clinical care are meeting the distinct needs of different athlete populations and this should be a target for future research.

## Data Availability

The datasets generated and/or analysed during the current study are not publicly available as ethics has not approved the sharing of data in this manner.

## Abbreviations

AIS: Australian Institute of Sport
NSO: National Sporting Organisation
GHQ-28: General Health Questionnaire
K-10: Kessler 10
WEMWBS: Warwick-Edinburgh Mental Wellbeing Scale
PGSI: Problem Gambling Severity Index
MDRS: Male Depression Risk Scale
AUDIT: Alcohol Use Disorders Test
EDEQ: Eating Disorders Examination Questionnaire
